# Two Cases of Malignant Colonic Obstruction in Which the Use of a Long Cap Enabled Successful Guidewire Passage

**DOI:** 10.14309/crj.0000000000001851

**Published:** 2025-10-13

**Authors:** Nobutaka Doba, Kosuke Shibayama, Shinzo Abe, Daiki Sakuma, Masanobu Someya, Kazuto Komatsu, Shin Maeda

**Affiliations:** 1 Department of Gastroenterology Yokosuka City Hospital, Yokosuka City, Kanagawa Prefecture, Japan; 2 Department of Gastroenterology, Yokohama City University Graduate School of Medicine, Yokohama City, Kanagawa Prefecture, Japan

**Keywords:** malignant large bowel obstruction, guidewire passage, self-expandable metallic stent, transanal ileus tube, long cap

## CASE REPORT

**Figure 1.**
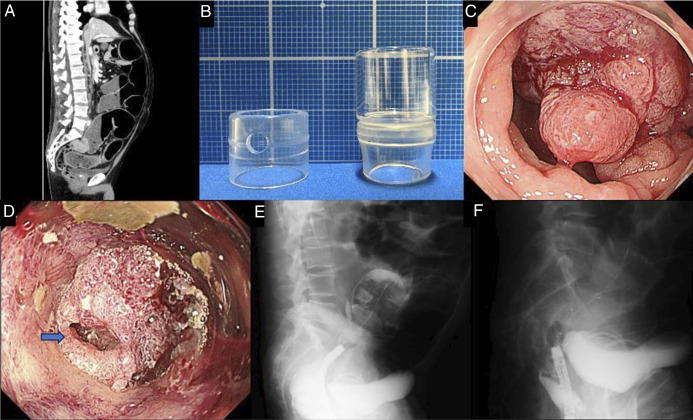
Clinical images of malignant large bowel obstruction because of rectal cancer and the endoscopic procedure using cap-assisted technique. (A) Pretreatment axial abdominal computed tomography image showing marked colonic dilatation proximal to the obstructive lesion in the rectum, suggestive of malignant large bowel obstruction. (B) Endoscopic caps used during the procedure. Left: short cap (4-mm protrusion; D-201-13404, Olympus, Tokyo, Japan); right: long cap (12-mm protrusion; MH-463, Olympus). (C) Endoscopic view of the anal side of the tumor using a short cap. The lumen is not identifiable. (D) Endoscopic view of the anal side of the tumor using a long cap. The lumen is more clearly visualized, and scope alignment is facilitated. Arrow indicates the lumen. (E) Fluoroscopic image after successful guidewire placement across the stricture. (F) Fluoroscopic image following deployment of a self-expandable metallic stent (SEMS), showing adequate expansion across the stenosis.

A 56-year-old man presented with malignant large bowel obstruction (MLBO) because of rectal cancer. Emergency colonoscopy with a short transparent cap (4-mm protrusion) revealed a circumferential tumor, but the lumen was poorly visualized, and guidewire advancement failed after 20 minutes. Switching to an endoscope with a long cap (12-mm protrusion) improved visualization and enabled guidewire passage within 4 minutes, allowing successful self-expandable metallic stent placement (Figure 1).

**Figure 2.**
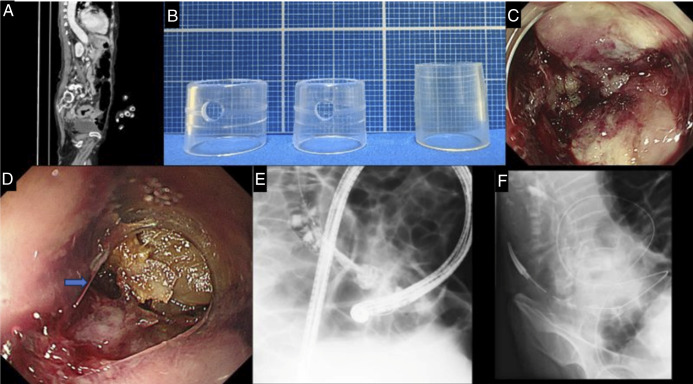
Clinical course of malignant large bowel obstruction because of sigmoid colon cancer and management using cap-assisted endoscopy and transanal decompression tube. (A) Pretreatment axial abdominal computed tomography image showing marked colonic dilatation proximal to the obstructive lesion in the sigmoid colon, suggestive of malignant large bowel obstruction. (B) Endoscopic caps used during the procedure. Left: short cap (4-mm protrusion; D-201-12704, Olympus); center: short cap (4-mm protrusion; d-201-13404, Olympus); right: long cap (7-mm protrusion; Space Adjuster, TOP, Tokyo, Japan). (C) Endoscopic view of the anal side of the tumor using a short cap. The lumen is not identifiable. (D) Endoscopic view of the anal side of the tumor using a long cap. The lumen is clearly visualized, facilitating scope alignment. Arrow indicates the lumen. (E) Fluoroscopic image after successful guidewire placement across the stricture. (F) Fluoroscopic image following placement of a transanal decompression tube, showing decompression of the dilated colon.

An 81-year-old woman with MLBO from a sigmoid tumor presented with hematochezia and anemia. Endoscopy with 2 different short caps (both with 4-mm protrusion) failed over 76 minutes because of poor visualization and colonic angulation. A long cap (7-mm protrusion) enabled clear lumen identification, allowing guidewire passage in 3 minutes. Because of the angulation, a transanal ileus tube was placed^1,2^ (Figure 2).

In both cases, long caps improved endoscopic visualization and guidewire control, facilitating successful decompression where short caps failed.^3^ The extended protrusion of long caps keeps the scope tip away from the mucosa, preserving the field of view and aligning the scope axis—especially useful in severe stenosis or angulated segments. While not without limitations, long caps may offer a simple, effective adjunct in technically challenging MLBO cases.^4,5^ Further studies are warranted to validate their broader use.

## DISCLOSURES

Author contributions: N. Doba: conceptualization, writing—original draft preparation, writing—review and editing, visualization; K. Shibayama and M. Someya: data curation; K. Komatsu and S. Maeda: supervision; S. Abe and D. Sakuma: investigation. N. Doba is the article guarantor.

Financial disclosure: None to report.

Informed consent was obtained for this case report.

## ABBREVIATIONS:

MLBO: malignant large bowel obstruction
SEMS: self-expandable metallic stent
